# Data describing the effects of Induced knockout of Heterogeneous nuclear ribonucleoprotein K in mouse skeletal muscle satellite cells

**DOI:** 10.1016/j.dib.2024.110576

**Published:** 2024-06-12

**Authors:** Haixia Xu, Yuxi Wang, Yijia An, Menghan Zhu, Xiaofang Cheng, Cencen Li, Pengpeng Zhang, Yongjie Xu

**Affiliations:** a College of Life Science, Xinyang Normal University, Xinyang 464000, China; b Institute for Conservation and Utilization of Agro-Bioresources in Dabie Mountain, Xinyang Normal University, Xinyang 464000, China

**Keywords:** Skeletal muscle satellite cells, Proliferation, Induced knockout, RNA-seq, Regeneration

## Abstract

HnRNPK, a prominent member of the heterogeneous nuclear ribonucleoprotein (hnRNP) family, is widely expressed in mammalian tissues and plays a crucial role in animal development. Despite its well-established functions, limited information is available regarding its role in skeletal muscle development and regeneration. To elucidate the functional role of hnRNPK in skeletal muscle, we utilized *Pax7^CreER^; Hnrnpk^LoxP/LoxP^* (*Hnrnpk* pKO) mice as a model, isolated primary mouse skeletal muscle satellite cells (MuSCs), and induced hnRNPK knockout using 4-OTH. Transcriptome sequencing was performed on four distinct groups: *Hnrnpk* pKO MuSCs undergoing proliferation for 24 h (ethanol 24 h) and 48 h (ethanol 48 h) after treatment with ethanol as the control, as well as *Hnrnpk* pKO MuSCs undergoing proliferation for 24 h (4-OHT 24 h) and 48 h (4-OHT 48 h) after treatment with 4-OHT as the hnRNPK-induced knockout group. The RNA sequencing data was generated using the Illumina HiSeq 2000/2500 sequencing platform. The raw data files have been archived in the Sequence Read Archive at the China National Center for Bioinformation (CNCB) under the accession number CRA015864. This data article is related to the research paper “Deletion of heterogeneous nuclear ribonucleoprotein K in satellite cells leads to inhibited skeletal muscle regeneration in mice, *Genes & Diseases* 11: 101,062, DOI: 10.1016/j.gendis.2023.06.031”.

Specifications Table

**Table:** 

Subject	Biological Science, Cell Biology.
Specific subject area	Skeletal muscle development and regeneration*.*
Type of data	Table, Figure. Raw, Analyzed, Filtered, Processed.
Data collection	*Hnrnpk* pKO MuSCs were treated with 4-OHT to induced knockout hnRNPK. MuSCs proliferation 24 and 48 h after the treatment, RNA was extracted, and RNA-seq libraries were generated. High-throughput RNA sequencing was performed by paired-end sequencing using Illumina HiSeq 2000/2500 (Illumina, USA). *Hnrnpk* pKO MuSCs treated with control, ethanol *vs. Hnrnpk* pKO MuSCs treated with 4-OHT.
Data source location	Xinyang Normal University, Xinyang, Henan Province, China*.*
Data accessibility	***Please note*** Repository name: CNCB BioProject. Data identification number: CRA015864 Direct URL to data: https://ngdc.cncb.ac.cn/gsa/search?searchTerm=CRA015864 Instructions for accessing these data: Free access
Related research article	Yongjie Xu, H. X., Xiaofang Cheng, Nuo Chen, Yaling Wang, Yueru Wang, Jiahua Guo, Yueqian Zheng, Mengjia Zheng, Chunyu Du, Cunzhen Zhao, Cencen Li Pengpeng Zhang (2024). Deletion of heterogeneous nuclear ribonucleoprotein K in satellite cells leads to inhibited skeletal muscle regeneration in mice. Genes & Diseases, 11, 101,062. DOI: 10.1016/j.gendis.2023.06.031.

## 1 Value of the Data

• The dataset contains the transcriptomes of MuSCs proliferation growth after induced knockout of hnRNPK, serving as a reference for studying hnRNPK's roles and downstream genes.
• The dataset provides key information for a comparative analysis of differentially expressed genes between the MuSCs and hnRNPK knockout MuSCs.
• Analysis of gene expression changes in proliferation growth of MuSCs and hnRNPK knockout MuSCs will help investigate the importance of hnRNPK in self-renewal and fate of MuSCs.
• In addition, the dataset can be used to examine the functional analysis of genes regulated by hnRNPK in MuSCs proliferation, and also it could provide very useful information to reveal the mechanism of action of hnRNPK in mouse muscle regeneration.

## 2 Background

A growing body of research on hnRNPK indicates its significance in animal development, cell proliferation, and differentiation via transcriptional and post-transcriptional regulation [1]. Nonetheless, there is a paucity of studies on its involvement in skeletal muscle development, and the downstream target genes remain unidentified. To address this gap, we generated *Hnrnpk* mKO by utilizing *Hnrnpk^LoxP/LoxP^* in conjunction with *Myf5*-Cre mice. The *Hnrnpk* mKO mice exhibited mortality prior to 18.5 dpc, with surviving 17.5 dpc mice showing significant absence of muscle tissue (unpublished data), underscoring the essential role of hnRNPK in skeletal muscle development. Subsequently, we generated inducible satellite cell specific knockout mice utilizing *Hnrnpk^LoxP/LoxP^* and *Pax7^CreER^* mice. The induced knockout of hnRNPK in mice severely impeded muscle regeneration. Mechanistically, the interaction between hnRNPK and *Cdkn1a* mRNA was implicated in these processes [2]. However, considering the complexity functions of hnRNPK, its target gene is not only *Cdkn1a*, but may also regulate MuSCs growth through other target genes. The induced knockout of hnRNPK in MuSCs was used for RNA sequencing to obtain differentially expressed genes by altered expression of hnRNPK. The resulting transcriptome data can be useful to identify potential downstream genes of hnRNPK that involved in skeletal muscle development and regeneration*.*

## 3 Data Description

**Fig. 1:**
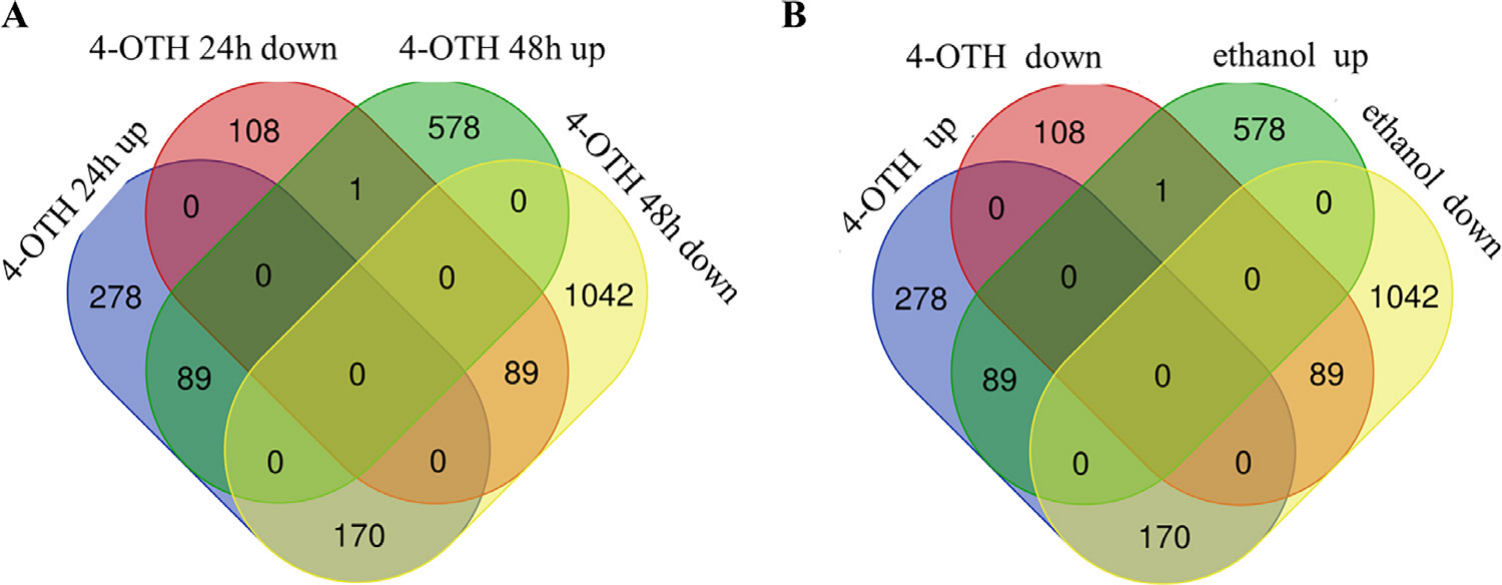
Intersection Venn diagram of differentially expressed genes in MuSCs. (**A**) Genes in MuSCs proliferation 24 h and 48 h treated with 4-OHT. (**B**) Genes in MuSCs proliferation growth treated with 4-OHT and ethanol.

The transcriptome data were produced from four different *Hnrnpk* pKO MuSCs groups, including proliferation 24 h after treated with 4-OHT (4-OHT 24 h), proliferation 24 h after treated with ethanol (ethanol 24 h), proliferation 48 h after treated with 4-OHT (4-OHT 24 h), and proliferation 24 h after treated with ethanol (ethanol 48 h). The raw sequence read data were deposited at Sequence Read Archive database of CNCB BioProject (https://ngdc.cncb.ac.cn/gsub/) under the accession number CRA015864. SRA accession numbers for 12 transcriptome data are CRR1111118 (4-OHT 24h-1), CRR1111119 (4-OHT 24h-2), CRR1111120 (4-OHT 24h-3), CRR1111121 (4-OHT 48h-1), CRR1111122 (4-OHT 48h-2), CRR1111123 (4-OHT 48h-3), CRR1111124 (ethanol 24h-1), CRR1111125 (ethanol 24h-2), CRR1111126 (ethanol 24h-3), CRR1111127 (ethanol 48h-1), CRR1111128 (ethanol 48h-2), and CRR1111129 (ethanol 48h-3) respectively. The raw and filtered read data were summarized in Table 1. Based on the mapping of the filtered reads on mouse reference genome GRCm38.p4 (mm10), the number of differentially expressed genes (DEGs) are obtained and represented in the Venn diagrams (Fig. 1). In total 735 and 1973 DEGs were identified from 4-OHT 24 h and 4-OHT 48 h compared to ethanol 24 h and ethanol 48 h control, respectively. On the other hand 2192 and 2392 DEGs were found in 4-OHT treatment and ethanol treatment during *Hnrnpk* pKO MuSCs proliferation growth. Table S1, Table S2, Table S3 and Table S4 are a list of tables representing all DEGs in *Hnrnpk* pKO MuSCs proliferation 24 h treated with 4-OHT, *Hnrnpk* pKOMuSCs proliferation 48 h after treated with 4-OHT, *Hnrnpk* pKO MuSCs proliferation growth treated with 4-OHT, and *Hnrnpk* pKO MuSCs proliferation growth treated with ethanol.

**Table 1:** Summary of RNA sequencing data.

Sample ID	Total Reads	Total Bases	Q20 Bases	Q30 Bases	Q20 Rate(%)	Q30 Rate(%)	GC Content(%)
4-OHT 24h-1	28,613,248	4291,987,200	4139,271,835	3914,602,197	96.4418	91.2072	49.4618
4-OHT 24h-2	49,271,364	7390,704,600	7203,942,158	6923,113,388	97.473	93.6733	49.261
4-OHT 24h-3	52,243,486	7836,522,900	7637,165,247	7336,533,504	97.456	93.6198	49.1844
4-OHT 48h-1	58,605,234	8790,785,100	8560,466,656	8222,193,232	97.38	93.532	47.9708
4-OHT 48h-2	47,323,688	7098,553,200	6922,615,960	6652,599,118	97.5215	93.7177	47.9489
4-OHT 48h-3	60,316,966	9047,544,900	8829,328,904	8484,222,781	97.5881	93.7738	48.8092
ethanol 24h-1	54,948,030	8242,204,500	8042,325,478	7727,833,071	97.5749	93.7593	48.7937
ethanol 24h-2	55,642,380	8346,357,000	8126,773,656	7793,845,887	97.3691	93.3802	48.1114
ethanol-24h-3	56,441,370	8466,205,500	8254,812,819	7934,839,596	97.5031	93.7237	47.7042
ethanol 48h-1	61,192,398	9178,859,700	8958,908,052	8625,951,152	97.6037	93.9763	49.5231
ethanol 48h-2	58,015,642	8702,346,300	8425,945,730	8089,652,860	96.8238	92.9594	50.4766
ethanol 48h-3	57,364,076	8604,611,400	8399,212,312	8078,999,119	97.6129	93.8915	49.1663

## 4 Experimental Design, Materials and Methods

### 4.1 Animal used

All animal experiments in this study were approved by the Institutional Review Board of Xinyang Normal University (Xinyang, China). Mice were hosted on a 12 h light/12 h dark cycle (8:00AM to 8:00PM) at humidity of 50∼70 % and 22 ± 2 ℃ ambient temperature with free access to food and water. *Hnrnpk ^LoxP/LoxP^* mice were generated with the aid of Cyagen Biosciences Inc. (Suzhou, Jiangsu, China) in a C57BL/6 genetic background. Genotypes were identified by PCR analysis as previously described [3]. Skeletal muscle satellite cell-specific *Hnrnpk* knockout mice were generated according to the strategy as follows. Briefly, inducible mouse model *Pax7^CreER^* (The Jackson Laboratory, stock no. 012,476) mice were crossed with *Hnrnpk ^LoxP/LoxP^* mice to obtain *Pax7^CreER^; Hnrnpk^LoxP/+^* mice, which were crossed with adult *Hnrnpk ^LoxP/LoxP^* mices, and the *Pax7^CreER^; Hnrnpk^LoxP/LoxP^* male mice were considered to be the conditional homozygous knockout mice (*Hnrnpk* pKO), and the littermates *Hnrnpk ^LoxP/LoxP^* mice were used as the wild-type (WT) control. Mice were genotyped by PCR using standard protocols.

### 4.2 MuSCs isolation and culture

MuSCs were isolated using type I collagenase and dispase B digestion as previously described [4]. Briefly, the hind limb skeletal muscles from the *Hnrnpk* pKO mice were collected, minced, and digested in 2.5 ml of collagenase/dispase solution (10 µg/ml collagenase B, 2.4 units/ml dispase B in PBS) for 1 h. The digestions were stopped with RPMI 1640 medium containing 20 % fetal bovine serum and centrifuged at 1000 g for 10 min. Then the cells were seeded on Matrigel-coated dishes and cultured in growth medium containing RPMI 1640 medium, with 20 % fetal bovine serum, 2.5 ng/ml bFGF, 0.5 % CEE, 1 % GlutaMAX, 1 % NEAA, and 1 % penicillin-streptomycin at 37 °C with 5 % CO_2_. The medium was changed every 2 days. MuSCs were used for analysis after purification by 2–3 times of pre-plating. To induce MuSCs differentiation, the culture medium was replaced with DMEM supplemented with 2 % horse serum (Gibco, USA) and 1 % PS at 80 % confluence. To induce the knockout of hnRNPK in *Hnrnpk* pKO MuSCs were treated with 40 nm 4-hydroxytamoxifen (4-OHT) (Sigma) for 48 h, and treated with ethanol as control. Cells were then used for the analysis after removal of the 4-OHT or ethanol.

### 4.3 RNA-Seq library preparation and analysis

RNA was isolated from hnRNPK induced knockout MuSCs and control MuSCs at proliferation 24 or 48 h after 4-OHT or ethanol treatment using RNAisoPlus reagent (TaKaRa, Japan). The qualified total RNA was further cleaned up by using RNAClean XP Kit (Beckman, Krefeld, Germany) and RNase-Free DNase Set (QIAGEN, Hilden, Germany). After passing through the quality inspection, the RNA-Seq library was constructed using the VAHTS Stranded mRNA-seq Library Prep Kit for Illumina® (Vazyme, NR602–02), and sequenced on the Illumina Hiseq 2000/2500 NextSeq with paired-end sequencing in Shanghai Bohao Biotechnology Co., Ltd. After prefiltering the raw data by removing the joint sequence and low-quality reads, the preprocessed reads were conducted to align the mouse genome GRCm38.p4 (mm10) using Hisat2 estimated by (version: 2.0.4) alignment software for genome mapping [5]. The expression level of each gene was normalized by FPKM (Fragments per Kilobase of exon model per Million mapped reads) using StringTie software (version 1.3.0) [6]. The gene differential expression was determined using EdgeR ver. 3.40.2 [7], and the statistical analysis of differential expressed gene (DEG) was calculated by DESeq2 ver.1.12.4 software with a Wald's test [8]. Genes exhibiting a log2Fold Change ≥ 1.0 or log2Fold Change ≤ −1.0 and Q-value ≤ 0.05 were considered to be significantly differentially expressed in the testes derived from 4-OTH group compared to in ethanol control groups, and growth 48 h group compared to 24 h group.

## Limitations

Not applicable.

## Ethics Statement

All animal experiments in this study were approved by the Institutional Review Board of Xinyang Normal University (XYEC-2021–011) and carried out in accordance with the National Institutes of Health guide for the care and use of laboratory animals (NIH Publications No. 8023, revised 1978)*.*

## CRediT authorship contribution statement

**Haixia Xu:** Methodology, Data curation, Writing – original draft. **Yuxi Wang:** Methodology. **Yijia An:** Methodology. **Menghan Zhu:** Methodology. **Xiaofang Cheng:** Supervision, Formal analysis. **Cencen Li:** Supervision, Formal analysis. **Pengpeng Zhang:** Supervision, Formal analysis. **Yongjie Xu:** Conceptualization, Funding acquisition, Writing – review & editing, Validation.

## Data Availability

RNA-seq of hnRNPK knockout and WT MuSCs in different stages of cell proliferation (Original data) (China National Center for Bioinformation (CNCB)).

## Appendix Supplementary materials

Image, application 1

Image, application 2

Image, application 3

Image, application 4
